# Precarious engagements and the politics of knowledge production: Listening to calls for reorienting hegemonic social psychology

**DOI:** 10.1111/bjso.12609

**Published:** 2022-12-20

**Authors:** Geetha Reddy, Amena Amer

**Affiliations:** ^1^ School of Psychology and Counselling Open University Milton Keynes UK; ^2^ School of Human Sciences, Faculty of Education, Health and Human Sciences University of Greenwich London UK

**Keywords:** decolonial, knowledge production, precarity, social psychology

## Abstract

In this paper, we invite psychologists to reflect on and recognize how knowledge is produced in the field of social psychology. Engaging with the work of decolonial, liberation and critical psychology scholars, we provide a six‐point lens on precarity that facilitates a deeper understanding of knowledge production in hegemonic social psychology and academia at large. We conceptualize knowledge (re)production in psychology as five interdependent ‘cogs’ within the neoliberal machinery of academia, which cannot be viewed in isolation; (1) its epistemological foundations rooted in coloniality, (2) the methods and standards it uses to understand human thoughts, feelings and behaviours, (3) the documentation of its knowledge, (4) the dissemination of its knowledge and (5) the universalization of psychological theories. With this paper we also claim our space in academia as early career researchers of colour who inhabit the margins of hegemonic social psychology. We join scholars around the world in calling for a much‐needed disciplinary shift that centres solutions to the many forms of violence that are inflicted upon marginalized members of the global majority. To conclude, we offer four political‐personal intentions for the reorientation for the discipline of hegemonic social psychology with the aim to disrupt the politics of knowledge production and eradicate precarity.

## INTRODUCTION

Knowledge production within hegemonic social psychology (and indeed within many other disciplines) is unequal and built upon engendering precarity within the field, yet this is little acknowledged when considering how to create a more inclusive and diverse field (Bacevic, 2021; Bou Zeineddine et al., in press; Gordon, 1985; Hendricks & Moghaddam, 2020; Hiemstra & Billo, 2017; Robinson, 2022). While numerous scholars have challenged hegemonic psychology and its sub‐disciplines for its theories, methods and approaches being embedded in specifically Western thinking and standards (Adams & Salter, 2019; Bou Zeineddine et al., 2022; Kessi, 2019; Martín‐Baró, 1986; Readsura Decolonial Editorial Collective, 2022a; Sinha, 1998), the reflections and reorientations offered by these scholars often lies on the periphery. Much of ‘whitestream’ academia (Grande, 2008, p. 233), within which the hegemonic psychology discipline operates, therefore, continues to fail to reflect upon how research is designed, data is collected and analysed and thus how, by whom, about whom and for whom knowledge is produced. Psychological narratives on peoples from the Global South have historically depicted racist views (Bhatia, 2002), where the Other is constructed as inferior or problematic in what is also referred to as epistemological violence (Teo, 2010). The foundations of such narratives continue to be woven into contemporary understandings of those that are viewed as the Other or alternate to ‘WEIRD (Western, Educated, Industrialised, Rich and Democratic)’ populations (Henrich et al., 2010; p.19) from which generalizations about psychology are often made. This lack of reflection and action towards uprooting these foundations continues to maintain, reinforce and reproduce structures of inequality, which reverberate within our societies. As social psychologists who study and make claims about the experiences, thoughts, feelings and behaviours of individuals who are entrenched in different socio‐political‐cultural worlds, it becomes fundamental we take stock of this and consider the asymmetry of power in knowledge production and how it perpetuates precarity. Not only is this our point of departure for this paper, it is also our call to our readers.

Whilst several delineations exist within the discipline of social psychology, some voices speak louder and reverberate longer than others forming what we and other scholars have named hegemonic whitestream psychology. The discipline's positivist roots and experimental turn led by its desire to be legitimized as a natural science^1^ is a modern, mainly North American steering of the field that has been rendered invisible yet made hegemonic (Farr, 1991; Lubeck, 2000; Teo, 2013). The Cartesian belief “I think, therefore I am” that is associated with WEIRD psychology is one of the ways that individualist lifeways that are free from material constraints are presented as natural standards in hegemonic psychology (Readsura Decolonial Editorial Collective et al., 2022b). This positivist, individual‐focused perspective does not apply to all of Western social psychology as can be seen in the development of European theories such as Social Identity Theory (SIT; Tajfel & Turner, 1979) and Social Representations Theory (SRT; Moscovici, 1984) that centres group level phenomena, as well as in the rise of critical social psychologies in the UK and US (Parker, 2007). However, Western psychology's narrow application of these theories results in context often being unaccounted for or used solely as a variable in research despite context being central to both these theories (Deaux & Martin, 2003; Huddy, 2002; Moscovici, 1984; Spears, 2001). As such, the spread of psychology across the globe has in fact resulted in an exportation of predominantly North American and West European psychological concepts, assumptions, approaches and methods (Bhatia & Priya, 2018) and adoption of North American standards so as to gain credibility and social status (what is referred to as ‘scientistic mimicry’) (Martín‐Baró, 1994; p.15). These concepts, theories and standards have come to be seen as ‘universals’, rather than as distinctly North American or West European psychological phenomena and has led to an active silencing and suppressing of other knowledge from other parts of the world (Canham et al., 2022; Osei‐Tutu et al., 2022; Ratele & Malherbe, 2020; Segalo et al., 2015). Hegemonic psychology is therefore *imperialist*, *individualist* and *universalist* in its orientation and actions (Readsura Decolonial Editorial Collective et al., 2022b). These perspectives that are also androcentric, ethnocentric, heteronormative and ableist reflect the way in which processes of knowledge production are embedded within global systems, structures and processes of power.

In writing this paper, we do not claim to make new or novel points, rather, we join scholars around the world in calling, once again, for a much‐needed disciplinary shift that centres solutions to the many forms of violence that are inflicted upon marginalized members of the global majority. This need to repeat this call speaks volumes to the way in which these concerns and injustices continue to be overlooked and ignored in order to maintain the narrow boundaries of hegemonic psychological interpretation that speaks from a global minority perspective. We offer our understanding of precarity as a lens to help us make sense of epistemic injustice in its different forms. We cast this light on academic knowledge production to make sense of how Western, imperialist, whitestream academia creates and engenders precarity. We draw examples from whitestream psychology to contextualize our points not by any means to limit our arguments to the discipline but rather to use cases familiar to us and readers of BJSP. Social psychology cannot be disconnected from the broader issues within academia and we nest our arguments within the understanding that the discipline of social psychology reflects and contributes to the precarities of academia. We are of course, well aware that we continue to work in these spaces we critique and we will continue to work with colleagues who desire to ameliorate conditions that are set upon us. We invite you to join us and fervently hope that this article, one day, ages out. That colleagues who read this article in the future read about the ways that hegemonic academia is structured with shock and horror because unequal worlds cease to exist.

## CRITICAL APPROACHES AND CALLS TO DECOLONIZE

We note first the work that has been and continues to be done in more critical engagements within the discipline and its practices for knowledge production. Critiques of hegemonic (social) psychology are not new (cf. Billig, 2008; Gergen, 1973, 1989; Guthrie, 1976/2004; Harré & Secord, 1972; Parker, 2007; Teo, 2006). The field's push to locate well‐being and emotions solely in the mind of the individual (and thus easily identifiable through laboratory experiments) has been cautioned against because minds are not ‘black boxes’ but rather thoughts are historically and socially constituted and communicated (Moscovici, 1984, p. 15). That the mind and society are not inherently separate (Jovchelovitch, 1996) and sensitivity to historical and political contexts is essential in deepening our understanding of the human psyche (Cornish, 2004) is both a recognition of how interconnected humans are and a rejection of hegemonic psychology's neoliberal narrative. Reductionist views within psychology have been challenged consistently, such as how we reify categories (Gillespie et al., 2012; Hopkins et al., 1997), understand collective behaviour and action (Templeton et al., 2018; Nair & Vollhardt, 2020) and study identities (Chryssochoou, 2003; Hammack, 2008; Marková, 2003), especially race (Hook & Howarth, 2005; Reddy & Gleibs, 2019; Richards, 1997; Tizard & Phoenix, 2002), sexuality (Hubbard & Hegarty, 2014; Kitzinger et al., 1992; Salvati & Koc, 2022) and gender (Morgenroth & Ryan, 2018). Discursive psychologists have shown the power that analysing talk holds in understanding what constitutes knowledge (Durrheim & Dixon, 2005; Potter & Billig, 1992; Seymour‐Smith, 2017), troubling the notion that only positivist experimental research is valid psychological science. Qualitative psychologists have interrogated the assumptions on ‘good’ research practices and have advocated for reflexivity to be fundamental to the research praxis (Lazard & McAvoy, 2020). Indeed, we see the power of reflexivity in Lukate (2022a) honest engagement and reflection of her role in the reproduction of social categories that are embedded within a hegemonic and Western lens in her own research. Questions underpinning knowledge production as it pertains to teaching environments (Kello & Wagner, 2017), citizenship (Andreouli, 2019), poverty and inequality (Sheehy‐Skeffington, 2019), religion (Coyle, 2008) and refugee migration (Mahendran et al., 2019) amongst many other issues that concern social psychologists, provoke what is readily accepted as the status quo in hegemonic psychology. Above all, scholars have urged us to confront the reality that social psychology has not been value neutral and that social psychologists have in fact perpetuated oppressions such as racism (Sambaraju & McVittie, 2021), anti‐Blackness (Phoenix, 2022) and sexual harassment (Young & Hegarty, 2020). Several critical psychologists have called for an expansion of the discipline by advocating for a societal psychology (Howarth et al., 2013) that develops new theoretical perspectives and methods to understand rapid societal change (Smith et al., 2019) and to mobilize for social change (McGrath et al., 2016). We are also encouraged to do ‘bad psychology’ (Grzanka & Cole, 2021) so as to produce an engaged, transformational social psychology that uses an intersectional analysis to reduce inequalities, promote social justice and centre epistemic inclusion (Phoenix, 2022; Settles et al., 2019).

More critical and Indigenous approaches such as Liberation Social Psychology in Latin America (Burton & Kagan, 2005), Filipino Psychology or Sikolohiyang Pilipino (Enriquez, 1993; Pe‐Pua & Protacio‐Marcelino, 2000), African psychologies (Nwoye, 2015; Ratele, 2017a, 2017b), Kaupapa Maōri Psychology (Rua et al., 2022) or Islamic psychology (Ali‐Faisal, 2020; Seedat, 2021), have argued that hegemonic psychology not only undermines the establishment of community, but can in fact support and strengthen unjust social systems, emphasizing the ways in which barriers in society affect the thinking and actions of individuals. As such, critical approaches and Indigenous psychologies shift away from mainstream understandings of psychological phenomena. These psychologies with a clearer anticolonial focus instead explore the role of power and history when understanding identities as relational and intersecting and as capable of change and transformation (Kessi, 2016; Kessi & Boonzaier, 2018). They posit that Western hegemonic psychology not only leaves out much of the world's population in its understanding and theorization and erases key nuances but imposes particular worldviews, which narrow and subvert alternative knowledge and research methodologies. Not only do they draw on tools that challenge hegemonic traditions of the researcher‐participant relationship by aiming to establish a sense of community between the researchers and participants, including them in the research process, and centring knowledge from within the research context being explored, they also stress the power differentials in the production of psychological research (Broesch et al., 2020; Fine et al., 2021; Kessi, 2018; Rua et al., 2022).

Distilling these different arguments, decades of scholarship from critical social, liberation and community psychologists often in and from Africa, Latin America, Asia and Oceania has outlined the effects of coloniality of knowledge and being on colonized peoples in the development of hegemonic psychology (Fanon, 1967/1952; Hakim et al., 2022; Kessi, 2019; Martín‐Baró, 1986; Martín‐Baró & Sloan, 1990; Memmi, 1965; Mignolo, 2011; Ndlovu‐Gatsheni, 2021; Ratele, 2019; Rua et al., 2022; Sinha, 1998). Coloniality refers to “long‐standing patterns of power that emerged as a result of colonialism but that define culture, labour, intersubjective relations and knowledge production well beyond the strict limits of colonial administrations” (Maldonado‐Torres, 2007, p. 243). Coloniality and modernity, two sides of the same coin, endures even after reclamation of colonized lands^2^ as mental colonization (or displacement of local knowledge; Ngũgĩ wa Thiong'o, 1986) and colonial mentality (or collective self‐hatred; Biko, 1978; Fanon, 1967/1952). Coloniality also reflects “an ongoing overvaluation of people of European descent in the modern global order” (Readsura Decolonial Editorial Collective, 2022a; p10) and echoes what Tema Okun (2022) refers to as ‘White supremacy culture’—a project of colonization and conditioning, which elevates and gives weight to whiteness over others. Calls for decolonizing Psychology (Adams et al., 2015; Barnes & Siswana, 2018; Decolonial Psychology Editorial Collective, 2021; Kessi, 2019; Macleod et al., 2020; Readsura Decolonial Editorial Collective et al., 2022a, 2022b; Seedat & Suffla, 2017) need to be heeded urgently because hegemonic psychology is a “superspreader of modern/colonial individualist lifeways” (Readsura Decolonial Editorial Collective et al., 2022b; p.260),

Thus, while these works and scholars provide social psychology with important considerations to reflect on and engage with, there is little space and acknowledgement of their contributions within hegemonic spaces. Instead, much of it exists within the margins, reflecting the inequality of knowledge production (Bou Zeineddine et al., 2022; Readsura Decolonial Editorial Collective et al., 2022a). Such inequity in whose knowledge comes to be recognized and which kinds of knowledge are allowed to take up space within the mainstream is also closely connected with experiences of precarity. In situating the problem of the politics of knowledge in our understandings of precarity, it allows us to shed light on how the exclusion of certain types of knowledge and knowledge holders within our discipline ensures the maintenance and dominance of hegemonic social psychology. We also use the opportunity of contributing to this special issue to bring these discussions into mainstream psychology so that we may (re)invigorate conversations on inclusion, diversity and social justice that have occupied many sub‐disciplinary journals and conferences of the British Psychological Society.

## PRECARIOUS WORLDS

Psychological scholarly focus on precarity as an important phenomenon that warrants our attention is not new (Fine, 2015; Hodgetts et al., 2016), but within hegemonic psychology these issues lie on the periphery even as they have garnered significant attention in other disciplines (Introduction to this special issue) Coultas et al., 2022. We present our understanding of precarity so as to show how we observe the contours of knowledge production in hegemonic social psychology as‐a‐representation of whitestream academia. We have developed our understanding of precarity using a six‐point lens drawing from scholarship within and outside of psychology, an interdisciplinary method we believe is necessary to adopt in eradicating social injustices that do not neatly lie in the realm of particular disciplines.

Firstly, we take a systems‐centred approach looking at the machinery of academia so as to dismantle how, in its current form, it creates and upholds precarity in academia through its particular ways of knowledge production. Whilst we recognize the importance of conceptualizing the precariat as a social class that is marked by insecurity and uncertainty for its strength in rallying political power (Standing, 2011), we wish to centre the precarious‐making ways of institutions. Seen in this light, academia can be viewed as a machinery that, like many labour institutions, partakes in the reproduction of race, gender, nationality and ableism‐based oppressions. When we take this approach, we can see that people are made precarious under certain conditions, and liberated from precarity (or some elements of precarity) in others, what Deshingkar (2019) refers to as the making and unmaking of precarious subjects. We draw from Söderström's (2019) point on *structural disempowerment* to talk about how particular ways of knowledge reproduction are built into the hierarchical and gridlocked systems that purposefully redirect power in service of privileged minority.

Secondly, we use a relational approach to looking at exchanges of power that take place within these systems so as to understand how power lies with certain peoples and is taken away from others. As Ahmed (2007) reflects on what it means to be a non‐white body within white institutional spaces, she notes that histories affect who and how space is taken up as “institutional spaces are shaped by the proximity of some bodies and not others: white bodies gather, and cohere to form the edges of such spaces” (p. 157). Thus, by looking at relations, rather than isolated individuals and comparing organized wholes (Elcheroth et al., 2011), we are able to identify the structures in which power lies, and investigate what this power looks like, who wields this power, and over whom and in service of who. By connecting contemporary power relations to colonial control, what Nkrumah (1965) conceptualized as *neocolonialism*, we identify spaces, actions and policies as sites of control over political and economic resources. Even as we focus on structures, our critique is still directed towards people who uphold these power structures. When we view a group of people as the precariat within an institution, we trade an understanding of the role of actors and results of their actions for a passive, individualizing view of the problem. In this relational approach, we name not only how systems are set up, but who they are set up by and who they are set up for. We are guided by this perspective on precarity to think through the ethical responsibilities of the practitioners of the discipline when envisioning the future of the field.

Thirdly, we draw from Fine's (2015) *epistemology of precarity* to direct attention to the ways that policies produce disruption in the lifeworlds^3^ of those engaged with precarity and how this disruption furnishes us with wisdom. This wisdom provides us with a particular way of viewing the world that may not be visible to those who do not experience precarity or live on the margin, and one's experience of precarity may not necessarily be reflected in another's. To this end, we draw from our own experiences throughout the paper, as insiders belonging to the very groups that we are researching with but also as outsiders being researchers in the whitestream academic institutions that we have studied and work(ed) in. This is our *locus of enunciation* (Grosfugel, 2016) and we share our embodied knowledge and reflect on how these knowledge take shape in relation with others. We do this explicitly to challenge the zero‐point epistemology that is endemic to hegemonic psychology that often results in the hypervisibility of scholars of colour and scholars from the Global Souths and the invisibility of White scholars and scholarship. Therefore, our understanding of precarity is one that is rooted in each scholar's locus of enunciation that in turn provides us with insights.

Like many critical scholars, we refer to the Global South(s) as an orienting lens, rather than a geographical positioning, to centre the marginalization and colonization that has systematically excluded and historically unrepresented colonized peoples, their knowledge and their scholarship (de Sousa Santos, 2015; Spivak, 1988). This is not to say that all communities in the Global Souths share the same struggles as reflected in our decision to refer to this orientation in the plural (cf. Montiel & Uyheng, 2022), or that decolonization processes alone will result in equitable societies. Homogenizing cultures (and nation states associated with those cultures) has been a facile way of understanding the Other (Sinha, 1996). An understanding of how pre‐colonial hierarchical structuring of societies, such as the caste system in South Asia (Nair & Vollhardt, 2020), continues to enact violence on marginalized communities in the Global South is important in exposing the ways in which knowledge produced by Indigenous communities remains erased by academics from those Global Souths countries. Geetha speaks as a scholar from the Global Souths working within Global North institutions who has had their voice controlled in the spaces that they have lived and worked in. Yet they have benefited from the ways their ancestors built and maintained power for those like themselves at the expense of others deemed less worthy. Amena speaks from a position where her visible Muslimness has been used to justify questions of her legitimacy within, and contribution to, academic spaces. Yet being born, brought up and educated in the Global North, she has benefited from an ‘insider’ experience not afforded to others. This paper, birthed from alchemising our experiences, reflects both our privileges as relative successes of the system that allows us to take up space as well continues to marginalize and silence scholars from the Global Souths.

Fourthly, we focus on collective agency so as to locate particular bubbles that sustain and nourish marginalized folks in the academy. With this focus, we also redirect the conversation away from pathologizing precarity as a condition that needs to be solved by each individual. Because precarity is endemic and built into the system, its solutions need to be collective in its conception and application, and accessible to all. We, therefore, are in conversation with scholars past, present and future in (re)aligning ourselves in service of collective abundance, rather than individual scarcity. Standing (2015) calls for those who experience precarity to be transformative in their actions and desirous of a new system of distribution of wealth. Whilst we take pains to detail the ways the knowledge production in hegemonic social psychology and academia precarizes scholars, we also wish to recognize where resistance is taking place. Precarity can provide a liberatory framework (Fine, 2015) that directs those focused on eradicating it. Therefore, this point in the lens of understanding precarity is focused on identifying collective refusals of the restrictive structures and power hierarchies.

Our fifth point on precarity is its embodied affect. Precarity inhabits spaces beyond work (Söderström, 2019). It infiltrates various aspects of marginalized scholars' lifeworlds that affect the way we navigate everyday life. For example, loneliness amongst activists stemming from the social pain of dislocation as they battle institutionalized precarity (Emejulu, 2021) can also be extended to academics who are forced to take up academic positions in places where they have little social networks. Scholars also experience precarity in building romantic, familial and kinship relationships when there is little stability in their different life worlds (Theo & Leung, 2022). Women disproportionately experience care‐led affective precarity when they stay in full time employment in academia (Ivancheva et al., 2019). Therefore, we also locate precarity in the bodies and minds of scholars who are experiencing precarity. This is to say that our theorization of precarity is that it is not an identity, or a psychological trait. It is an embodied state of feeling, being and thinking that one can slip in and out of when in connection with precarious‐making structures.

Lastly, we take the view that whilst precarity is a universal, and politically induced condition (Butler, 2009, 2012), it does not affect everyone in the same way because of how power operates to regulate humanity accorded to each individual and how vulnerability is unequally distributed (Ruti, 2017). For example, job precarity and constricted academic freedom affect women of colour (and other gender minorities) more in UK higher education (Albayrak‐Aydemir & Gleibs, 2022; Blell et al., 2022a). Therefore, we observe how precarity affects everyone who engages with it, and highlight how multiple, intersecting oppressions collide within precarity to engulf folks already experiencing racism, sexism, homophobia, transphobia and white supremacy.

We will next explore these anchors of knowledge production within academia using this six‐point lens not only to highlight the multidimensionality of precarity within social psychology and, therefore, academia but also to make manifestly clear how epistemic violence, made up of intellectual imperialism (the imposition of knowledge based on WEIRD research) and epistemological violence (Readsura Decolonial Editorial Collective, 2022b) politicizes knowledge production and practices, and perpetuates precarity.

## THE POLITICS OF KNOWLEDGE PRODUCTION

Audre Lorde (1984) warned us that the available methods of knowledge production are inadequate to critiquing academia and will reproduce the patriarchal whitestream systems that we are trying to change. We, therefore, conceptualize knowledge (re)production in this paper with the awareness that whilst many of us engage with theories of power, we may not know how to translate this knowledge into praxis and that our negotiations we have made with structures thus far may have simply enabled us to enter and survive in academia, rather than change it (Chandrashekar et al., 2018). Therefore, with humility and through the six‐point lens we explained above, we draw out five key interdependent elements that make up the machinery of knowledge production and reproduction in psychology. Namely, we consider (1) social psychology's epistemological foundations rooted in coloniality, (2) the methods and standards the discipline uses to understand human thoughts, feelings and behaviours, (3) the documentation of the discipline's knowledge, (4) the dissemination of discipline's knowledge and (5) the universalization of psychological theories. We stress that while we will take each of these elements individually to delve deeper and explain to our reader their significance, they should not be viewed in their silos, as independent entities in and of themselves. Rather, we must view this machinery in its whole, as containing interdependent and interconnected cogs that function in relation with one another to maintain the status quo and turning together to keep the neoliberal machinery of academia alive, productive and profitable.

### Colonial epistemological foundations

Psychology's, and indeed social psychology's, colonial epistemological foundations set the disciplines up for creating precarity. Critical scholars have carefully documented Western modes of knowledge production across the social sciences and humanities to note how these modes continue European projects of colonial expansion (Mignolo, 2011; Said, 1978; Spivak, 1988). Hegemonic psychology's curation of knowledge founded on racist ideologies on human difference (Richards, 1997, 2009) and beliefs about taming the natives (Bhatia, 2002) has led to an epistemic exclusion of knowledge from elsewhere in the world (Settles et al., 2021). That imperialists created a hierarchy of knowledge where Indigenous communities were forced to follow and adhere to rules, customs and ways of their colonizers and endogenous wisdoms were subjugated is important in understanding this politics of knowledge (Ndlovu‐Gatsheni, 2021). Because the effects of colonization are not limited to the discrete periods when colonizers violently eradicated whole communities, ways of being and knowledge, what is referred to as coloniality, we see this subjugation of knowledge continue to manifest today in social psychology (Bou Zeineddine et al., 2022). Therefore, the institutions (such as universities, academic societies and journals), which are the spaces in which the epistemic violence of knowledge production is perpetuated come to be the very centre of the coloniality of knowledge (Bell, 2018; Sacchi et al., 2021). We see how the question of ‘whose knowledge counts?’ is answered for example, in the systematically excluded and historically marginalized scholarship produced by scholars in and from the Global Souths and those who seek to challenge hegemonic whitestream psychology.

This question overlaps with important social psychological and socio‐political questions about the politics of recognition in other contexts and the very real damage that can be caused by non‐recognition and denial of belonging (see Amer, 2020; Amer & Obradovic, 2022; Hopkins & Blackwood, 2011). For the above‐mentioned scholars, this marginal and precious space of existence can be both lonely and liberating, reflecting two points on the lens of precarity we highlight above of embodied effect and collective agency. Loneliness comes by way of choosing to engage in criticality and decoloniality within one's scholarship which comes at the cost of being ostracized from the mainstream and creating a sense of vulnerability to one's position within the academy. Liberation, however, can emerge through the fruitful (co)production of knowledge whilst taking up space in the margins, working alongside other scholars that support and hold up each other and their work (Fine, 1994; Hooks, 1990). We return to this point in more detail later and go on to call for due recognition of their work as rigorous intellectual praxis and for an acknowledgement of their humanity to be accorded—something that is all too often lacking in hegemonic whitestream psychology and only further strengthens its colonial foundations.

Yet another facet of hegemonic psychology's colonial epistemological foundation is its zero‐point epistemology, or what Malherbe and colleagues refer to as the “racism of the zero‐point” (Malherbe et al., 2021). In other words, this perspective of “the world looks like this from here”, expected of scholars in the Global Souths, exists because “the world looks like this from nowhere” for hegemonic psychology (Nagel, 1986). This release from having to locate one's positionality, in essence a modern individualist abstraction from context, produces a sense of entitlement and access to everywhere (Smith, 2012). More dangerously, and under the guise of analytical distance and objectivity (a point we return to later), this perspective can lead to false equivalences such as presenting both Black rage against police brutality or terroristic violence in support of white supremacy as equally problematic (Decolonial Psychology Editorial Collective, 2021).

This radical abstraction of context leads to, and stems from, psychology's neoliberal focus. Such is this circular relationship that not only focuses on the individual and consideration of society only as relates to that individual (looking at the ‘I' rather than interacting systems), it also promotes an entrepreneurial understanding of the self and fixates on individual growth (Adams et al., 2019). Psychological processes are often theorized at the individual level in hegemonic social psychology (Rizzoli et al., 2019). Hegemonic psychology's promotion of the neoliberal self has in fact spilled over to how it understands people in the Global South(s) (Bhatia & Priya, 2021). Within the discipline as it manifests in Euro‐American institutions, precarity is also a condition that is created through the avaricious expansion of short‐term teaching and research contracts that foist insecurity and vulnerability upon early career scholars (as discussed in Albayrak‐Aydemir & Gleibs, 2022). This condition in turn promotes competition and a scarcity mindset that is perfectly comfortable in the neoliberal, for‐profit paradigm that universities actively construct and maintain. The bar continues to be set much higher for new entrants intending to work in academia, yet in the same breath institutions speak of ramping up diversity and inclusion efforts to widen access to participation in university. Junior teaching staff in modern (otherwise known as post‐92) institutions in the UK and liberal arts colleges in the US have incredibly high teaching loads that leave little room for research and little energy for protests. Precarization within academia forces people to think solely as individuals; individuals who need to secure the limited permanent contracts, individuals who need to publish more first author pieces in the handful of journals deemed as ‘high‐impact’ and ‘REF^4^‐worthy’, individuals who need to be principal investigators in large scale grants. Precarity forces compliance, silence and overwork and produces imposter syndrome (Bayly, 2022). These stress‐inducing pursuits come with hefty price tags that affect vulnerable folks more than others. Insufficient responses to the COVID‐19 pandemic have further demonstrated that university managerial practices hurt women of colour, particularly early career scholars (Blell et al., 2022b). We are pushed to find individual solutions—such as taking wellness days off or signing up for mindfulness courses—for endemic collective problems. Fanning the flames of precarity in academia demands a centring of egos and an individualizing of problems and solutions, and we refuse to hold people experiencing precarity responsible for the conditions that got them there.

Whilst these systemic issues affect all scholars in Euro‐American institutions, scholars on the margins (relegated there because they have not been accommodated for in this neoliberal machinery, nor has the academy been created with these scholars in mind) have struggled with these same systemic issues for decades. Global Souths scholars have battled and continue to battle inequalities rooted in coloniality and contemporary systemic procedural and distributive injustices in material, human and social–political capital that are further amplified by Northern hegemonies in social, institutional, disciplinary, economic and political systems (Bou Zeineddine et al., in press). Senior scholars of colour in Global North institutions who publish prolifically and are leaders in their fields are also not exempt from the stresses of precarious employment, as exemplified by recent cases in the US (Kraus, 2022; Robertson, 2021). The recent strikes in UK and US higher education have been a clarion call for those who are not personally affected by the deplorable state of affairs that sustain precarious working conditions. Precarity in Euro‐American academia is also not limited to research and teaching staff—many cleaners, library staff, security staff and administrative support staff battle precarity (for example, Justice for LSE Cleaners, 2018). Knowledge production in higher education today is built on the backs of many forced into precarious working conditions and eradicating this cog would require us to be epistemologically disobedient (Mignolo, 2009; Sacchi et al., 2021).

### Whitestream methods and standards

Whilst social psychologists have previously publicly disavowed racism (Letters from 40 psychologists, 1990), their role in maintaining racist research practices that reproduce whitestream psychology is a more recent acknowledgement (American Psychological Association, 2021). Those which are considered normatively good practices within psychology, and academia at large, such as adherence of (narrow definitions) of rigour and validity actually produce epistemic and structural barriers (Abo‐zena et al., 2022; Grzanka & Cole, 2021; Lau, 2019), showing that methods and standards within hegemonic practices of knowledge production perpetuate precarity from both a system‐based and relational perspective as outlined above. We are at once reminded of Fanon who asserted that “methods devour themselves” (Fanon, 1967/1952; p. 14). The dominance of experimental methods in leading social psychology journals like European Journal of Social Psychology (EJSP) are representative of the narrow epistemological, methodological and geographical focus of the discipline (Rizzoli et al., 2019; but see Imhoff et al., 2018 on new directions for EJSP). The promotion of particular methods also results in application of set methodological standards onto different and diverse methods that instead require standards of their own. A clear example of this is the response to the so‐called ‘replication crisis’. Rather than being an issue that is universal to (all) Psychology/(ies), the replication crisis has come about because of a particular way of doing hegemonic research and is specifically relevant and unique to quantitative methods. Yet, the standards of replicability, validity and reliability that are heavily embedded within quantitative standards become imposed on all methods in the name of pushing for Open Science (Prosser et al., 2021). In other words, hegemonic Open Science movements seek to fix problems that are created because hegemonic psychology takes a universalist, expansionist perspective on its theorization of human behaviour but does not seek to eradicate epistemic injustice (Goh et al. Goh et al., forthcoming). Its goals are limited, and continue to perpetuate precarity (but see Open and Collaborative Science in Development Network's manifesto for visions of an Open and Collaborative Science, 2022).

Several epistemic violence occur when attempts are made to correct issues pertaining to whitestream methods, in particular ‘data collection’ and research ethics. For example, in the push to uptake scholarship in non‐WEIRD countries in the name of ‘un‐WEIRD’‐ing psychology, many researchers engage in extractive practices, with little to no engagement with the communities from whom data is sought as a part of the research process (Broesch et al., 2020) what is referred to as *epistemic extractivism* (Grosfugel, 2016). Thus, research continues to be done on, rather than with, Otherised peoples and has continued the imbalance of power in how knowledge is produced with a disregard for the value of local and personal knowledge. Indigenous communities have suffered and continue to suffer under the (dis)guise of research, highlighting how coloniality masquerades as scientific and technological advancement (or modernity) (Smith, 2012). Research ethics evaluations devoid of critical analyses of coloniality, racism, ableism, homophobia and transphobia reproduce white normativity and dominance (Dirth & Adams, 2019; Myser, 2003; Tuck & Guishard, 2013). Material and epistemic precarities are often ignored when attempting to increase inclusivity in research (Papoulias & Callard, 2022). Academic imperialism also instructs whose lives are researched and whose lives get preserved (Fine, 2018) and this power imbalance can also be observed in the lack of reflection and psychological studies on the role that one's privilege has ‘in producing, sustaining, naturalizing and framing injustice’ (Stoudt et al., 2012; p.179). Many ‘data collection’ practices echo psychology's colonial epistemic foundations which still continue to reverberate today, often exploiting the most vulnerable in society.

Another example of a key issue that is endemic to hegemonic psychological methods that adds to the precarity of participants is the notion of ‘imperial translations’ where methods are rooted in colonial, Eurocentric thought (Ndlovu‐Gatsheni, 2019) and analyses are defined through the lens of Western stereotypes and a privileged way of understanding the world (Fine, 1994). We discuss this in detail in the section on the universalising of psychological theories, but its relevance to methods must be noted. We argue that these imperial translations have implications for how the data is co‐constructed with individuals, how it is analysed, and how it is reported. Who gets to research certain communities? Who gets to be in the position of researcher? Who gets to attain this position of privilege to research these communities? How does the researcher decide which methods to use? These questions reflect the epistemic positioning and epistemic injustice experienced by marginalized scholars and enacted by scholars in positions of power in academia (Bacevic, 2021). This is to say that psychologists' researcher positions are integral in the creation of knowledge about human behaviour and interactions. Engaging with the discomfort that this reflection of our positionalities brings is an important part of understanding the role we play in (re)creating precarity for our participants (Reddy, 2021a) and as such it becomes integral that one begins to interrogate their role in epistemic positioning and injustice.

Thus, in considering the contexts of precarity where race, ethnicity, religion, class, migration patterns and culture create unequal social worlds for individuals, we call for a questioning of the role of the researcher when engaging with the ‘researched’ and highlight the importance of acknowledging researcher positions in relation to those being researched. Whitestream psychology creates the figure of a detached observer (Mignolo, 2009) and has valorized the notion of distance between researcher and participants, insisting on objectivity as a standard of rigour. In doing so, it dismisses the reality that we, as members of society, as scholars, as psychologists are not isolated from social conventions (Gergen, 2001) and overlooks important considerations of sameness in the research context as adding depth and richness to our interpretations (Bhopal, 2010; Phoenix, 1994). In fact, scholars of colour who take up ‘insider’ positions by doing research with communities they belong to and do research with are often burdened with the requirements of foregrounding their identities (Lukate, 2022b) as a means of declaring bias rather than as sources of knowledge. It, therefore, only further embeds an insistence on objectivity as a standard of rigour and popularizes the myth that research by researchers who are cloaked in a perceived notion of neutrality or objectivity is more valid. In the absence of intersectional analyses, whitestream epistemologies, methods and standards reinforce racism, ableism and patriarchy (Rizvi, 2022). The practice of reflecting on one's ‘insider‐outsider’ positions intersectionally and taking action to not inflict harm upon participants needs to be central to psychological methods.

### Documentation of knowledge

How we document the knowledge encountered become sites of further epistemic violence within the process of knowledge production and these violence persist in the way in which the publication process works. Journal impact factors are often not entirely reflective of quality as highlighted by self‐citation practices in niche sub‐fields (Brumback, 2009), yet funding frameworks, notably in the US and the UK focus on assigning limited research funding by assessing candidates based on their documentation of knowledge in ‘high‐impact’ journals. Many scholars favour international journals over niche journals or more accessible repositories that cater to the communities that knowledge is gained from. Relatedly, poor citational practices reinscribe power to white and WEIRD scholars, erasing the labour of and contributing to epistemicide of Otherised scholars (but see #CiteBlackWomen; Ahmed, 2016; de Sousa Santos, 2015; Mügge et al., 2018; The Critical Ethnic Studies Radical Citation Practice Challenge, 2015) 5. More profoundly, epistemic violence is also inflicted when psychological scholarship on conflicts suffers from both‐sideism and features a striking absence of marginalized scholars who experience the material precarities of the violence in addition to the epistemic violence (Hakim et al., 2022).

The demands of a ‘publish or perish’ culture, rooted in the colonial epistemological foundations described above, push data collection down explicitly extractive and unethical routes of obtaining as much information as easily and efficiently as possible in order to churn out publications. These pressures leave little to no space for more collaborative knowledge productions between the researcher(s) and participants, which requires an investment of both time and funds. Increased competition and demand by journals including the steady rise in the number of studies published in journals has further perpetuated this. For example, articles in journals such as the European Journal of Social Psychology and Journal of Personality and Social Psychology contained an average of 1.95 and 4.43 studies each in 2015–6, respectively compared with 1.55 and 1.75 studies in the early 90 s (Kruglanski et al., 2016). This increase in standards expected of funded research and publications and indeed the recent publication acceleration and fast‐track schemes, which promise editorial decisions within weeks such as that provided by Taylor & Francis^6^ further favour and reward those with access to funds. While this may (albeit indirectly) be as a result of increased demands, expectations and pressures within academic roles (Kruglanski et al., 2016)—for example, recently promoted professors in social psychology have a much higher publication record than those in other psychological fields highlighting the particular competitive pressures in our own field (Valla, 2010)—it brings up an important question. *For whom* do we do research? While our research should be shared with our peers and colleagues both within and across our disciplines, what about our participants, without whom our research would not exist?

Documenting knowledge through collaborative engagements with participants (often referring to them as co‐researchers), centring care and solidarity and blending both research and action towards social justice has long been practised by psychologists committed to liberation and more recently, published in academic journals (Atallah & Dutta, 2022; Bell, 2016; Fine & Torre, 2019; Kessi, 2018; Torre, 2009). More importantly, these scholars have focused on disseminating knowledge co‐created with their fellow researchers in accessible ways that sit outside of hegemonic psychology's rigid curation practices. Knowledge is produced and documented outside of academic structures, by former scholars and scholars in exile from Indonesia, Syria, Turkey, Palestine, Middle East, Ukraine, amongst many other countries (Hünler, 2022; Parkinson et al., 2018; Theo & Leung, 2022), yet often these wisdoms do not make it to mainstream outlets highlighting how our colleagues who are experiencing gender, sexuality, religion, nationality‐based oppressions engage with precarity more acutely than others. ​​Intertwined with the epistemic violence of how knowledge is documented is the way in which knowledge is disseminated, an important aspect of knowledge production that we delve into next.

### Dissemination of knowledge

Hegemonic systems and structures for knowledge dissemination are created by and for a historically dominant minority echoing the way in which precarity, and indeed power, is maintained. This presents significant challenges and hurdles for Global Souths and Otherised scholars. One such challenge is the issue of language. With English being the default language for the majority of mainstream journals (Flowerdew, 2008), we see how non‐native English‐speaking scholars or indeed those who do not have sufficient command of the language to write in an academic style, are at a significant disadvantage compared to native speakers (Ferguson et al., 2011). Moreover, such standards and boundaries to knowledge production and dissemination results in the loss of important contributions, limiting the possibilities of alternative perspectives and echoes the colonial and imperialist project of hegemonic knowledge production (Bulhan, 2015). It perpetuates a culture within hegemonic psychology that puts a further burden on researchers on margins to “publish in English or perish in academia” (Bocanegra‐Valle, 2014, p.65), highlighting the precarity of their position within academia and the pressure to engage within specific hegemonic spaces in order to succeed.^6^ Indeed, as Bou Zeineddine et al. (2022) note, marginalized and Global South(s) social psychologists engage in a ‘coerced compliance’ to publish in and meet the standards of internationally recognized journals more often than not based in the US or Europe, while simultaneously cognizant of the very real barriers they face. Depressingly, colleagues from the Global Souths are unable to access the very journals that their articles are published in because of high‐cost barriers and lack of institutional agreements between journals and low‐income institutions.

### Universalization of psychological theories

The last cog we highlight in the machinery of academia in perpetuating precarity and hegemonic knowledge production is its focus on universalizing theories developed based on a global minority. This notion of psychological universals benefits from the large volume of published psychological research taking place in WEIRD contexts and this pursuit of psychological universals has left a significant mark on the fabric of social psychology (Reicher, 2004). Whilst hegemonic psychology's ethnocentric perspective is more readily accepted in that its core assumptions and theoretical insights are more often than not drawn from data collected on WEIRD populations yet are widely applied to people and contexts beyond these ethnocentric boundaries (Bulhan, 2015; Henrich et al., 2010), its zero‐point epistemology furthers the idea of knowledge as universal (Mignolo, 2009). Founders of European theories such as Social Identity theory have been prudent in advocating for their theories as answers to racism for example (Phoenix, 2022). Many of these theories are not equipped to answer pressing social issues of today (Smith et al., 2019) and yet they are often used liberally as solutions to multi‐faceted contemporary societal challenges.

Research that did not conform to existing Western theories and models were considered exceptions that unfortunately left the theoretical bases unchallenged (Sinha, 1998). Psychologies from countries like India (Sinha, 1998) and China (Yang, 1997) have had to adopt hegemonic psychology's modes of knowledge production to be recognized as credible sciences. One response for integration and acceptance into mainstream psychology was for local psychologies in various societies to develop their own respective indigenous psychologies, with the hope that “they then be gradually integrated to form a genuine global psychology” (Yang, 1997; p. 70). Yet, this model of global psychology has not always been considered feasible because it has been viewed as continuing the hegemony of the West (Bhatia, 2002). Therefore, internationalization of psychological research and theories remains an imperialist endeavour that is both expansionist (that is, based on the idea that the right way of doing psychology needs to be given away to people outside of the USA and Western Europe) and assimilationist (that is, international work will need to be incorporated into mainstream Western centric psychology) (Adams, 2018). Indigenous psychologies are crucial, yet by classifying applied psychological work as indigenous psychology *only* the problem persists because when whitestream psychology fails to engage with such research, the insights that Psychology as a discipline can gain then diminish. Principally, forcing all psychologies and psychologists to fit a particular mould created by hegemonic psychology continues to perpetuate epistemic exclusion, expropriates power from Otherised communities and upholds the imbalance of power in knowledge production.

Whilst many psychologies outside of the tight boundaries of hegemonic psychology are thriving on the margins (Pe‐Pua & Protacio‐Marcelino, 2000; Ratele, 2017a), practitioners of hegemonic psychology have recognized the paucity of diverse viewpoints and have campaigned to recognize scholarship from Otherised peoples. When there exists a limit to who gets to frame the research questions in the field, this leads to an impoverished psychology (Sellers, 2022). This reckoning has led to an increase in the hiring of scholars from these communities as can be seen when Euro‐American institutions actively encourage applications from individuals with various protective characteristics under the banner of equality, diversity and inclusion. While well‐intentioned, this only signals the start of a long process of un‐doing and making‐right of the deeply violent and exclusionary practices of hegemonic psychology. Indeed, by focusing simply on the inclusion of different categories of people (such as hiring scholars from marginalized communities and/or the Global Souths), we can easily fail to account for a core principle of justice—that is the acknowledgement of harms and violence inflicted upon peoples Otherised across the globe.

Critiques of inclusion and diversity initiatives and practices state that whilst the recognition of the dominance of whiteness in our research and institutions is important, it has done little to alleviate the inequality in the access to and participation within academia (Ahmed, 2006, 2007). Such inclusion and diversity practices often entail adding more representation from communities that have been systematically excluded in academic spaces, without putting in place the measures necessary to ensure that this historical exclusion does not take a new form today or changing institutional structures that continue to alienate scholars from marginalized communities. This move requires Otherised scholars to perform their worthiness, and in some cases even outperform their colleagues, to be allowed into the ivory towers of psychology. In addition, their performance needs to showcase a diversity of thought without disrupting hegemonic psychology. This focus on diversifying curricula by simply adding more Black and brown scholarship rather than challenging the foundations of whitestream psychology is yet another manifestation of violence within academia. Thus, in focusing on inclusion through practices such as increasing the hire of non‐white psychologists, we are at best inviting people to this table of whiteness (Reddy et al., 2021). We are not changing the white supremacist institutional structures that continue to alienate and Otherise scholars from marginalized communities. Distressingly, such inclusion tactics result in ‘diversity clickbaiting’ where Otherised scholars who have joined this table are consigned to embodying diversity in university and end up being on the menu as seen in the cannibalizing of Black feminist scholarship (Bilge, 2020; p.317). Eradicating the barriers to Black and brown scholars' participation and scholarship in whitestream psychology is a necessary first step to take if psychology were to take its agenda on ensuring equitable representation in its institutions seriously (Dupree & Kraus, 2022). Much more needs to be done to truly counter the universalization of whitestream psychological theories, and more broadly, arrest precarity in academic knowledge production.

## REORIENTING SOCIAL PSYCHOLOGY

Several critical scholars have called for an imagination of a new university (Bell et al., 2020; Emejulu, 2018), a ‘new scholarly imaginary’ (Pickren & Teo, 2020; p.3) and an expansion in the scope of what is considered to be a Euro‐American centric social psychology (Kessi & Kiguwa, 2015; Sundararajan, 2014), what we refer to as whitestream hegemonic social psychology. We note that whitestream psychology is not only upheld and reproduced by white folks. It is a system of knowledge making that anyone can and does end up buying into, as shown in the five cogs above. Each of us can accept different levels of responsibility to upholding it, but the job of dismantling it has to be a collective project rooted in compassion, deep understanding and generous self‐reflection. We also register the unequal burden on scholars of colour, especially Black scholars, to not only articulate the problems that they experience, but also to find solutions for them. Therefore, we share some political‐personal^7^ intentions here for reorienting Social Psychology so as to uproot precarity and we invite others to think through with us and act upon in their roles as educators and researchers.

Before we discuss our intentions (that echo those of many who come before us), we want to take this opportunity to affirm our point on the pluriversality of social psychologies that our colleagues in community psychology have also called for (Sonn et al., 2022). The acknowledgement of multiple social psychologies in the discipline of social psychology is not new (Lubeck, 2000), yet psychologies not in service of hegemonic psychology have been relegated to the margins. This is not an argument for these alternative psychologies to be validated by hegemonic psychology. It is a call for an engagement with the margins, but equally important, a reflection of how the margins came to be and what our roles are in perpetuating these margins. The margins provide a ‘special vantage point’ to both critique the hegemony and also ‘envision and create a counter‐hegemony’ (Hooks, 1984; p.15). There is the productiveness of the margin in our abilities to be rid of ‘disciplinary decadence’ (Gordon, 2014) and discover possibilities in inter and transdisciplinary collaborations (Stenner, 2015). In reclaiming the term ‘marginalized’, we reassert our power, reject the passivity of this position and set ourselves up to thrive on these margins created by hegemonic social psychology. In rejecting the boundaries of the discipline constructed through these margins, we envision a lush rainforest. One does not know where this rainforest ends, and each organism in the rainforest exists in a symbiotic relationship with the other. Multiple social psychologies exist, thrive and feed into each other, not as a way of reproducing itself or appropriating one another but with the understanding that we are connected and our survival is dependent on these connections. Counter to the *discipline* of hegemonic psychology, which is focused on gatekeeping and governing boundaries, we see a rich forest of social psychologies nourished by diversity in epistemologies and locations of enunciations. The decay of whitestream psychology provides fertile ground for this jungle to thrive.

Our first intention is for social psychologists to engage in actions that eradicate epistemic injustice. There are several paths to building better worlds where epistemic justice and knowledge from the global majority can be centred. Epistemological decolonization is especially poignant when, for example, we saw Palestinian, Arab and allied social and political psychologists withdrawing from presenting at an international political psychology conference (Adra, 2021). They did so in protest of the fact that in spite of the conference's theme on the recognition of colonization, it effectively denied Palestine recognition as an occupied and colonized land in its programme, since most abstracts on the Israeli‐Palestinian context failed to make any reference to the context as being one of occupation, apartheid and settler‐colonialism (R. Saab, personal communication, October 11). Colonization is ongoing in Palestine, and we need to hold coloniality of knowledge and colonization of Palestinian and Indigenous peoples in Aotearoa New Zealand, Aztlán, Palestine, Papua New Guinea or Turtle Island at the same time. It requires us to think about and act upon the ways that we (un)knowingly perpetuate epistemic injustice. Malherbe et al. (2021) shared that a decolonial Africa(n) centred psychology does more than direct attention to settings and social actors that have been neglected by mainstream psychology. It is a transformational rethinking (or an unthinking) of hegemonic WEIRD science. A decolonial psychology requires us to not only appreciate Other ways of knowing as legitimate sources of understanding about the embeddedness and relationality of life but also recognizes the violence that whitestream psychology has wrought via investment in and refinement of modern/colonial individualist lifeways as a model for human life (Decolonial Psychology Editorial Collective, 2021). At a political‐personal level, this requires unlearning, relearning and engaging in acts of refusals that do not signal disengagement (Coultas, 2022). This paradigm shift also requires a decentring of existing standards of knowing and being in Psychology (Decolonial Psychology Editorial Collective, 2021). This reorientation is not just about acknowledging the politics of knowledge but actively changing how we produce and reproduce the different cogs of knowledge mentioned above. It is about continuing to “coax the demise of anti‐Black racist psychologies” (Suffla & Seedat, 2020; p.294) and aligning ourselves with epistemologies from the South (de Sousa Santos, 2015). It is about changing the ways we consume knowledge and also what we do with this knowledge. To this end, decolonizing psychology is an incomplete project as long as curriculum development and hegemonic institutional structures continue to profit in the ways that they do currently. An application of decolonial praxis on knowledge production requires a restructuring of not only the way universities teach psychology but also the way it benefits from it. This is the work of transnational collectives of scholars engaged in building solidarity and committing to liberate our minds and bodies from whitestream knowledge production even when it may benefit us in the short term.

Our second intention is for social psychologists to make space for complaint. Writing this article weighed on us for several reasons. Firstly, our complaints are laborious acts that we undertake in service of building pluriversal social psychologies that seek to eradicate precarity in the field. We have already noted the demands placed upon researchers of colour to explain, justify and validate themselves and their research. With the pressures of the academic job market and increased precarity that come with fixed‐term contracts, going against the mainstream and calling in those who not only interrogate our work on unfair grounds but also continue to foist precarity upon early career researchers and marginalized communities is a deeply uncomfortable position for us to be in. Secondly, and relatedly, we are deeply aware that complaint is a political act that fixes the problem onto the individual and not on the system that the complaint is directed at (Ahmed, 2021). Historically, colonized peoples have been seen as “problems instead of people who face problems” (Du Bois in Gordon, 2000, p.84; Mariátegui, 1988). In the neoliberal academy, the complainer *is* the problem. Whilst one may contend that systems for complaint exist within academia, historically excluded and minoritized academics bear the brunt of already deep‐rooted cultures around targeted silencing (see Blell et al., 2022a for a deeper exploration of this issue). What we hope this means for us as early career scholars (especially those marginalized within hegemonic academia) seeking permanent employment in institutions willing to do the heavy work necessary to manifest this rainforest of psychologies is that we find ourselves in good company within and out with academia. In making space for complaint, we are planting seeds for connection, organization and resistance (Deveci, 2019; Qureshi, 2019; Rosales & Langhout, 2019). What we intend for this to do is to reorient hegemonic social psychology so that it makes space for complaints and complainers who are challenging the precarization of labour and knowledge (re)production in psychology. Sara Ahmed argues that this is because complaints are also where one learns about institutional mechanics and how institutions reproduce precarity when they reproduce themselves (Binyam, 2022). Complaints thus allow us to remake institutions and structures to serve all and not just the privileged few.

Our third intention is for social psychologists to advance critical pedagogy skills that visibilise insider‐outsider research and teaching positions. Uncovering our complicities in the creation and maintenance of hierarchies of knowledge needs to be central to our research process. This excavation is often an emotional process as challenging able‐bodied, cis‐hetero patriarchal, White supremacy when creating a justice‐oriented psychology is unsettling, disruptive and deeply uncomfortable (Reddy, 2021b). Even so, there needs to be a shift beyond a recognition of individual privileges and land acknowledgements to a critique of institutional structures, uncritical application of hegemonic theories to communities in the Global Souths and erasure of Indigenous knowledge. Developing a critical pedagogy that enables us to confront our complicities in Others' suffering (Zembylas, 2019) is our intention for this reorientation so that we birth a critical collective consciousness that excavates our role in (re)creating precarious conditions. In developing a deeper understanding of our complicities, we need to learn how to articulate our insider‐outsider positions and teach our students how to do so well. Oguntokun (1998) and Fine & Weis (1998) illustrate how one can be considered an insider to the communities they are seeking to understand, whilst simultaneously being positioned as outsiders to these very communities. As early career scholars of colour, these experiences resonate with us because our qualitative and quantitative research projects have asked from us (unequally) to share and shed our life histories, ways of being and names. Indeed, our interests in studying the social psychology of race and racism and experiences of belonging stems from our own life experiences. We strongly believe that this makes our work richer, and not lesser for it. We are motivated by understanding the underpinnings of how society races the individual and how the individual responds to this racialization in their everyday experiences. Sharing ourselves and our own stories in the research processes allows for knowledge to be co‐constructed through collaborative and dialogical means. It is part of the wisdom we have gained from experiencing precarity within social psychology and in our everyday lives. No doubt our own experiences influence our research, our relationships with research participants and our analyses of the data. This is true for all researchers, but whitestream psychology actively polices this imposition of objectivity, applying limits to who can be seen to provide a clear account of psychological phenomena. Deeply interrogating one's positionality when engaging in research with communities other than those that one belongs to and is surrounded by reveals the precarity of address (Lukate, 2022a). We invite social psychologists to follow in the footsteps of Silva et al. (2021) and Coultas (2022) and write and reflect on their roles in knowledge production in academia and produce their own *testimonios* so as to amplify silenced voices and make space for decolonial methods and pedagogical praxes that challenge us and the field of social psychology.

Our fourth and last intention is one that captures the essence of the above three intentions to centre our relationships with one another as we traverse various social worlds. Butler (2012, p. 148) reminds us that “precarity exposes our sociality, the fragile and necessary dimensions of our interdependency”. Uprooting precarity within psychology means stepping away from individualizing problems and solutions and centring the social in social psychology (Kessi, 2016; Parker, 2007). It means developing ways that end epistemic violence and disrupting and dismantling hegemonic whitestream knowledge production and reproduction without reinscribing them. We are asking social psychologists to reorient the field away from individualism and to think about how we are in connection with each other; to change structures that demand and provide individual solutions because systemic change is too heavy a price for hegemonic psychology to pay. This process also involves recognizing and working through the tensions that manifest when building solidarity between groups of people with varying levels of privilege (Nadesan, 2019; Selvanathan et al., 2022). It requires us to rethink the pace in which knowledge is expected to be produced and recognize how disabled and Otherised scholars are often left in the wake of this academic culture of/on speed (Lau, 2019). How will we then work in (and with people living in) this wake to enact transformative justice (Burgess, 2022)? We are inviting social psychologists to mindfully co‐create spaces that support scholars and knowledge production from and with the Global Souths (Martin & Dandekar, 2022) and Otherised scholars in the Global North because our knowledge and liberations are tied up with each other. We are manifesting a new university, alongside Deanne Bell, Hugo Canham, Urmitappa Dutta and Jesica Fernández (2020), that will invite all of us to come together in critical solidarity to resist neoliberal knowledge production and that allows us to heal the wounds created by colonization and forged by precarity.

## FINAL CALL

Indeed, despite academia's reputation of liberal or even radical political ideologies, and more specifically social psychology's aim to understand and overcome inequality, much of the knowledge that is shared, reproduced and seen as central to disciplines reflects the dichotomy of who is worthy of being listened to as contributing to knowledge and who is (and can be) ignored as detailed in various sections of this paper. Viewing knowledge production within social psychology and academia using the six‐point lens of precarity allows us not only to see the role psychology has played in creating deeply unequal and violent worlds but to also (re)imagine future versions of the field that centres epistemic justice. In the face of this sobering capture of the bleak state of affairs in hegemonic whitestream psychology, we rely on a methodology of hope that is both critical and reparative to imagine alternatives to current engagements with precarity (Khan, 2022) with our four political‐personal intentions. We present precarity as merely the lens that allows us to see the elephant in the room. Our work is not done until precarity finds no water in these new worlds and we invite you to join us, and those who have come before us, to engage in the revolutionary labour necessary to eradicate precarity in social psychology and academia.

## AUTHOR CONTRIBUTIONS

**Geetha Reddy:** Conceptualization; formal analysis; project administration; visualization; writing – original draft; writing – review and editing. **Amena Amer:** Conceptualization; formal analysis; writing – original draft; writing – review and editing.

## ACKNOWLEDGMENTS

We would like to thank our friends and colleagues whose conversations have encouraged us and fed into this paper. Geetha would also like to thank fellow members of the Readsura Decolonial Editorial Collective for their generative discussions on coloniality and decoloniality over the years. Finally, we would like to thank the five anonymous reviewers, Clare and Johanna for their critical comments and encouragements to produce this piece as it stands today.

## CONFLICT OF INTEREST

All authors declare no conflict of interest.

## Data Availability

There is no data to be shared.

## Footnotes

^1^
What many in the West call science today is in fact a narrow, patriarchal project that uses a mechanistic reductionist mode created to exploit knowledge for the industrial revolution (Vandana Shiva in Beckett & Beckett, 2021).

^2^
We hold the importance of “Land back” decolonial movements focused in Aotearoa New Zealand, Turtle Island, Palestine or Aztlán as we discuss other material and epistemic ways that coloniality continues to manifest itself in our worlds.

^3^
The lifeworld is an intersubjectively shared space where “communities link the past, the present and the future through social memory, social representations and social identities” (Jovchelovitch, 2007; p.79).

^4^
REF or Research Excellence Framework is a research assessment process required to be undertaken by UK higher education institutions approximately every 7 years. One of its aims, as stated on the REF website, is to maintain a high standard of research and impact across disciplines and is key in determining how UK research funding is distributed by the UK government. It has, however, been widely criticized by many academic, not least for how universities often celebrate successes while simultaneously perpetuating a system of precarity and staff burnout (Sayer, 2014; Watermeyer & Derrick, 2022).

^5^
We would like to thank Dr Monique Guishard who, in her role as reviewer five, offered this point for us to reflect on and incorporate in our work.

^6^
Taylor & Francis (2022) is just one publisher, which has recently published an acceleration publication options, which allows researchers to pay for faster processing of their submitted papers. Further information on this can be found on their website.

^6^
We also wish to stress ‘publish and perish’ action‐reaction made visible by Martín‐Baró (1986), which puts already marginalized scholars in life threatening situations when they articulate the violence they face from oppressors.

^7^
We adopt Sivanandan's view that the political is personal produces a radical society that allows one to open oneself to the oppression of others and to act upon them in the process of building new communities of resistance that will tackle power, Capital and class, rather than the position that the personal is the political, which may produce radical individualism (Sivanandan, 2009).
